# Effects of *Promove – Saúde da Mulher* on anxiety, depression, social skills, and satisfaction: A single-subject design

**DOI:** 10.1186/s41155-022-00226-y

**Published:** 2022-07-14

**Authors:** Alexandre Aguiar Victuri, Alessandra Turini Bolsoni-Silva

**Affiliations:** 1 Programa de Pós Graduação em Psicologia do Desenvolvimento e Aprendizagem – Faculdade de Ciências - Universidade Estadual Paulista “Júlio de Mesquita Filho” (UNESP) – Bauru, Avenida Engenheiro Luiz Edmundo Carrijo Coube, 14-01 Vargem Limpa, Bauru, SP 17033-360 Brazil; 2 Departamento de Psicologia – Faculdade de Ciências - Universidade Estadual Paulista “Júlio de Mesquita Filho” (UNESP) – Bauru, Avenida Engenheiro Luiz Edmundo Carrijo Coube, 14-01 Vargem Limpa, Bauru, SP 17033-360 Brazil

**Keywords:** Anxiety, Depression, Women, Manual, Behavior therapy, Behavioral-analytic therapy, TAC, Clinical behavior analysis

## Abstract

Terapia Analítico-Comportamental (TAC) (in English, behavioral-analytic therapy, behavior therapy, or clinical behavior analysis) is a possible intervention for cases of anxiety and depression, but it still has much to advance in terms of efficacy and clinical utility evidence. This article aims to describe the effects of a semi-structured intervention in the model of TAC regarding anxiety and depression, behavior, complaints, and satisfaction indicators. Participants included three women with children, marital relationships, and work, aged between 34 and 41 with complaints compatible with anxiety and depression disorders. The intervention used *Promove – Saúde da Mulher* (PSM) (in English, Promote Women’s Health), which included 17 topics, most of them related to social skills. Instruments included the GAD-7 for anxiety, PHQ-9 for depression, IHS-2 Del Prette for social skills, a Complaints Checklist for monitoring clinical demands, and an Evaluation of the Therapeutic Process to describe clients’ satisfaction rates. The results from the standardized instruments were statistically analyzed via the JT method. All three participants showed reliable improvements in anxiety and/or depression, improvement in most complaints, and satisfaction with the intervention and its outcomes. Acquisition of social skills occurred in two cases. One participant dropped out and another one relapsed at follow-up. The efficacy criteria were used to evaluate the internal validity of the present study. The study collected the first evidences of outcome and satisfaction for PSM, thus enabling future investigations on the efficacy and clinical utility of this intervention program.

The prevalence of anxiety and depression disorders (American Psychiatric Association, 2013) is a worldwide public health problem. In Brazil, which ranked first in the prevalence of anxiety and third in depression in 2015, the situation is even more pressing (World Health Organization, 2017). The damage caused by these disorders is amplified if they occur simultaneously, which is fairly common (Cyranowski et al., 2012; Fernandes et al., 2018; Starr et al., 2014), since approximately 50% of people present both disorders simultaneously (Johansson et al., 2013).

From the point of view of behavior analysis, anxiety disorders involve a wide variety of respondent and operant contingencies, both verbal and non-verbal, in addition to establishing operations that increase the likelihood of anxious behaviors (Coêlho & Tourinho, 2008; Dymond & Roche, 2009). In this process, signals of aversive stimulation play an important role as triggers for anxious responses (Skinner, 1953; Zamignani & Banaco, 2005). Therefore, responses maintained by positive reinforcement decrease, which can be explained by the individual’s overexposure to aversive stimuli (conditioned suppression). We also observe an increase in avoidance and escape responses—especially when aversive stimuli are avoidable or controllable (Coêlho & Tourinho, 2008).

Based on the same theoretical approach, depressive disorders can appear as a result of three simultaneous or isolated processes: loss of availability of reinforcing stimuli, inefficiency of one’s repertoire to acquire reinforcers, and/or lower magnitude of stimuli’s reinforcing value for the individual (Abreu & Abreu, 2017). Dougher and Hackbert (1994) present other explanatory variables for depression, namely excessive uncontrollable punishment and unintentional reinforcement of depressed behaviors by close people. An example of the latter would be when a partner offers attention and support whenever their depressed wife cries or isolates herself. The same applies to anxious behaviors (Coêlho & Tourinho, 2008).

## Incidence of anxiety and depression among women

Anxiety and depression disorders are more often reported by women (World Health Organization, 2017), which could be explained by cultural (Correia & Borloti, 2011; Heise et al., 2019) and biological (Li & Graham, 2017; Noble, 2005) risk factors. Cultural factors include gender inequalities and assigned roles at home and work, such as attributing to women the responsibility for household chores and childcare (Correia & Borloti, 2011) as well as workplace discrimination (Heise et al., 2019). If analyzed from a functional standpoint (Fonseca & Nery, 2018), such factor may account for an increased rate of contact with signaled and controllable aversive stimulation (e.g., spouse’s requests related to meals evoke anxious behaviors, which leads to the preparation of meals) and uncontrollable aversive stimulation (e.g., gender-based violence resulting in depression). In addition, task overload competes with the access to important reinforcers (e.g., social life, leisure activities) for the woman.

## Interpersonal repertoire as a risk factor

Mahan et al. (2018) and Starr et al. (2014) have shown an important relation between interpersonal problems and the onset of anxiety and depression. From a clinical standpoint, an intervention aimed at improving interpersonal relationships can minimize the negative impact of risk factors that make women more vulnerable to these disorders.

Throughout psychotherapy, women can learn to behave toward changing anxiety- and depression-building contingencies. For instance, when being criticized by their spouses for not preparing the food, they can learn to defend themselves skilfully by stating that household chores must be split and negotiate task division. This can help them get collaboration from their spouses and lower task overload, even though they may occur in the medium or long term. Thus, we believe that teaching interpersonal repertoires capable of producing positive and negative reinforcers in family and work relations may lower anxiety and depression indicators given that such behaviors may help, on one hand, lower adversities present in these situations and, on the other, maximize access to positive reinforcers. In that sense, multiple interventions that support the treatment of individuals who present symptoms of anxiety and depression simultaneously are required.

## Evidence-based practice

Several options for the psychological treatment of simultaneous anxiety and depression have been documented in the literature, including internet-delivered cognitive behavior therapy (or iCBT, in Păsărelu et al., 2016), interpersonal and mindfulness therapy (Mahan et al., 2018), the online mindfulness-enhanced cognitive behavioral therapy (Kladnitski et al., 2018), and the behavioral therapy (Schwartz et al., 2019). All showed positive results (e.g., improvements in anxiety and depression scores, participant satisfaction) and limitations (e.g., low adherence, low replicability), and not all focus on interpersonal relationships, which may be closely linked to the onset of the disorders (Mahan et al., 2018; Starr et al., 2014).

## Behavioral-analytic therapy and evidence-based practice

Behavioral-analytic therapy (in Portuguese *Terapia Analítico-Comportamental*, hereafter TAC^1^) (Meyer et al., 2010) is a form of behavioral therapy (Skinner, 1953) which mainly aims to develop an individual’s behavioral repertoire, thus increasing access to reinforcers and lowering aversive stimulation present in their lives (Cassas & Luna, 2018). It is then compatible with a large portion of interpersonal demands of anxious and depressed individuals. However, considering the guidelines of the American Psychological Association (2006) for evidence-based practice in psychology, this form of intervention still lacks evidence of efficacy and clinical utility (Moraes & Silveira, 2019).

Evidence-based practice seeks to integrate three main elements to provide the best intervention for the service user: data from scientific studies, the clinician’s expertise with the intervention, and the client’s personal characteristics and preferences (American Psychological Association, 2006). To attend American Psychological Association’s guidelines, TAC needs to demonstrate its efficacy and clinical utility through empirical studies with different designs.

Efficacy studies are the first step in that direction as they test the internal validity of a given intervention, that is, whether it reaches the variables it proposes to (American Psychological Association, 2006; Durgante & Dell’Aglio, 2018). In a theoretical study on evidence-based practices in psychology, Durgante and Dell’Aglio (2018) proposed 17 fundamental and 17 desirable criteria to assess the intervention programs’ efficacy, in addition to 16 items to assess their effectiveness. The authors pointed out that fundamental efficacy criteria must be prioritized in studies with new intervention programs.

After analyzing the efficacy of an intervention, wider studies can be carried out to assess their external validity and clinical utility compared to other interventions (Durgante & Dell’Aglio, 2018). As this consists of a first TAC intervention study, we sought to approach the efficacy element in our analyses and investigations.

Moreover, Moraes and Silveira (2019) pointed out that clients’ satisfaction rates with the intervention also need to be further assessed by TAC interventions. The authors relate this demand with the third element of evidence-based practices, which concerns clients’ preferences and characteristics. The extent of satisfaction with both the therapy and therapist has been studied in other approaches such as Kladnitski et al. (2018) and Sidani et al. (2017), and also needs to be addressed by TAC, considering the American Psychological Association’s guidelines (2006). The present study aims to include this aspect in its search for evidence.

## Manualization of interventions

To increase the consistency and replicability of psychological interventions for specific audiences and demands, some authors resort to the development and application of intervention manuals (Zettle, 2020), which differ from rigidly structured protocols, as they point to possible guidelines rather than a highly structured intervention program, which might hinder the flexibility required in each case. Zettle (2020) highlighted the importance of single-subject designs for the construction of a flexible and process-focused evidence-based clinical practice. We point out that, according to the American Psychological Association (2006), interventions that enable adjustments and flexibility for each individual under treatment tend to produce better results than those from interventions that are rigid and insensitive to their characteristics and demands. When it comes to TAC, there are intervention manuals that have been developed by our research group to be applied with specific audiences and have shown promising results (Bolsoni-Silva & Fogaça, 2018; Ferraz, 2018).

Considering the scope of the present study, we can observe that the available manuals presented specific work contexts (e.g., marital life, parenthood) and may not cater for people with interpersonal problems in different areas of their lives. This is the case for women with anxiety and depression, who may have a high frequency of psychological problems related to marriage, parenting, and/or work (Levatti et al., 2018). The present study lies in this gap, as means to devise a program that seeks to cater for such demands, simultaneously.

A manual geared to this public is under development and takes into account the same methods but in multiple contexts. Additionally, it uses case formulation (Sturmey, 2014), which is a personalized study of the main characteristics of a given case, enabling the adaptation of the intervention to the demands of each client. The present study aims to describe the effects of a semi-structured intervention along the lines of TAC, *Promove – Saúde da Mulher* (PSM), for a sample of three women with anxiety and depression indicators, regarding indicators of anxiety and depression, behavior, complaints, and satisfaction, in addition to adding efficacy evidence to the field of TAC.

## Method

### Ethical considerations

This study was approved by the university’s Research Ethics Committee. Participants agreed to participate in the research and publication of its results by signing an informed consent form.

### Participants

Three participants were selected from the waiting list of a psychology training clinic located in the public institution where the research was carried out. To be able to join the waiting list for psychological treatment, participants had gone through screening interviews, carried out by psychology undergrad interns. After the interview, interns reported participants’ identification data and main complaints and submitted their applications to the clinic’s virtual platform. Screening reports were available to university interns, researchers, and professors. Based on the data available in these documents, participants were pre-selected by researchers, based on the DSM-V (American Psychiatric Association, 2013) and the researchers’ knowledge of anxiety and depression disorders. They were initially contacted by e-mail and/or telephone. The candidates had to meet the following inclusion criteria to take part: be within the ages of 30 and 50 years old, be a mother, be in a marital relationship, be working, have reported symptoms of anxiety and depression in the screening interview report, not having reported another psychological disorder in the screening interview report, and achieve cutscore (10 points) for anxiety and depression in the screening instrument at baseline 1.

### Brief case formulation

The following will present key information gathered from the case formulation (Fonseca & Nery, 2018) of all three participants.

Participant 1 was 36 years old, divorced from her first marriage, in a stable relationship, and living together with her fiancé. She had two children from her first marriage, a 10-year-old girl and a 16-year-old boy. The couple got married during the intervention period. She worked as a hairdresser in her own salon and as a teacher in a vocational school. She was identified in the screening report by the following statements: “complains of being very anxious” (anxiety); “does not feel safe with her partner, even though he has never given indications of any kind of cheating” (anxiety, excessive worrying); “experienced postpartum depression” (depression). This participant had not been on any psychiatric medications, but eventually took flower essences, which she claimed to calm her down.

Participant 1 came to therapy complaining mainly of physical symptoms of anxiety and aggressive behaviors directed at her children (especially her daughter) and her husband. She hoped that therapy would help her feel and behave more peacefully toward her husband. From her life history, we highlight deprivation of affection by her parents, their models of aggressive parenting practices, health problems of her first child, postpartum depression in her second pregnancy, and the discovery of her ex-husband’s extramarital relationship.

The contingencies in place at the beginning of therapy involved verbal aggression toward the husband and children in situations of high demand for her attention, maintained by a reduced demand; behaviors of excessive preoccupation and checking the well-being of her children in moments of physical separation from them, maintained by anxiety relief and her children’s attention; remedial behaviors after aggressions, maintained by reconciliation; and jealousy behaviors toward the husband, maintained by his attention or guaranteed fidelity. The sum of these behaviors produced complaints from the children and husband and made the domestic environment a context of unpredictability and aversiveness, in the form of criticism/complaints, which evoked anxiety and provided few reinforcers, thus leading to her depression.

The specific therapeutic goals for this participant were to boost positive interactions with her children and husband by increasing her repertoire of expressing praise, gratitude, and affection; to reduce aggressive behavior by increasing functionally equivalent socially skilled repertoires (Goldiamond, 2002; Sturmey, 1996), to self-control emotion and aggression; to reduce excessive worrying by increasing alternative repertoires for attention and altering rule control over what she considered to be a good mother; and to engage the client in pleasurable activities for herself.

Participant 2 was 34 years old, married, and had two children with her husband: a 9-year-old daughter and a 12-year-old son. She worked as a nursing technician and self-employed seller of candy and cosmetics. Shortly before the intervention, she stopped working as a nursing technician and focused on selling cosmetics. She was also an undergraduate student in pedagogy. She was identified by the following statements: “feels overwhelmed and stressed, taking her anger out on her family” (anxiety, irritability), “feels overwhelmed and ignored” (anxiety, tiredness/fatigue), “had postpartum depression after both pregnancies” (depression), and “currently takes antidepressants” (depression). This participant had a diagnosis of fibromyalgia and was taking medications with muscle relaxing effects (such as Velija 60 mg) for pain management, as well as antidepressants (such as nortriptyline, dosage not reported). Her prescriptions were changed several times throughout the research.

Participant 2 came to therapy complaining mainly of physical symptoms of depression, anxiety, fibromyalgia, aggressive behavior toward her children and husband, and task overload and conflicts in the work environment. She hoped that therapy would help her reduce her sense of emotional overload and stress, lower aggressive behaviors, improve her marital relationship, and improve her relationship with her children. From her life history, we highlight domestic violence between her parents, deprivation of affection, aggressive parenting models, controlling behaviors and threats from her husband, and postpartum depression in both pregnancies.

The contingencies in place at the beginning of therapy involved physically and verbally aggressive behavior with her children in situations of disobedience to house or school rules, maintained by their temporary obedience and avoidance of complaints from her husband; permissive behavior with her children in situations of greater deprivation of affection, maintained by an affectionate response of her children and behaviors of avoidance/forgiveness of conflicts through the use of sleep-inducing drugs. The sum of these behaviors produced further behavioral problems in her children in the medium and long term, complaints from the husband about the participant not performing domestic chores and continued accumulation of tasks, as well as associated feelings and behaviors of anxiety and depression resulting from these contingencies.

Specific therapeutic goals for this participant were to make the relationship with her husband more reinforcing by increasing her repertoire of expressing affection, to make him aware of her human rights and the difficulties she was facing by increasing her assertive repertoire to deal with her husband’s complaints and demands (expressing opinions, asking for behavioral changes); to increase collaboration of children and husband with household chores by increasing her repertoire of asking for help, praising, and thanking; and to minimize her children’s behavior problems by increasing her repertoire of establishing clear rules and providing reinforcing consequences for compliance.

Participant 2 only attended the first half of the study. After the midterm evaluation, excessive absences configured dropout.

Participant 3 was 41 years old, married, and had an 11-year-old daughter. She was a public employee in a health care institution. She was identified by the following statements: “she freezes when needs to perform activities and experiences sweating, dry mouth, and increased heartbeat when afraid” (anxiety); “she said she feels anxious at all times” (anxiety); “she said she is a crabby, nervous person and that her daughter ‘doesn’t deserve to have a nagging mother’” (depression, lack of self-esteem); and “she also reports difficulties in her social life as she makes excuses not to go out with people; she said she thinks a lot before doing any activity, and even gives up sometimes” (depression, escape and avoidance behaviors). This participant did not use psychiatric medications.

Participant 3 came to therapy mainly complaining of physical symptoms of anxiety and depression, household chore overload, and aggressive behaviors with her daughter and husband. She hoped that therapy would help her reduce anxiety and improve social interactions. From her life history, we highlight her parents’ aggressive and strict educational practices, her father’s punishment of her decision-making behavior, and financial difficulties she faced with her husband 9 years before she was called to join the study.

The contingencies in place at the beginning of therapy involved passive behavior, maintained by avoidance of verbal conflict and, at work, by the rule that she should be friendly outside the home; aggressive behavior, maintained by the husband and daughter’s intermittent collaboration in domestic chores; and perfectionist behavior maintained by rules and parental models of how a woman should run a household. The sum of these behaviors produced affective detachment from the daughter and husband, task overload, and large amounts of time spent on household chores, which lowered her availability for positively reinforcing activities, and elicited anxiety and depression respondents.

Specific therapeutic goals for this participant included increasing the rate of positive interactions with her daughter and husband by developing affection expression skills; balancing the distribution of household chores with her daughter and husband by developing the skills of making requests, negotiating chores, and giving praise and positive feedback when they were met; reducing the time spent on household activities by reformulating perfectionist rules; and reducing the frequency of fights by expanding her aggression self-control repertoire.

All three participants had complaints related to their children and husband at the start of the intervention. They struggled to express both positive and negative feelings, as well as split tasks equally at home. Many of the passive and aggressive behavioral patterns of participants, as well as their deficits in affection expression skills, may be related to the aversiveness-based parental educational practices they experienced in their childhood and adolescence, generating aggressive models, little behavioral flexibility, and associated negative feelings. Due to a lack of alternative repertoires, these have persisted throughout the participants’ adult life at high frequency (usually in negative reinforcing contingencies), but also producing significant punishments.

### Data collection design and procedure

We used an adapted multiple-baseline design across participants (Cozby, 2003), which contemplated a different number of pre-intervention assessments for each participant, besides the possibility of analyzing pre-intervention changes. Before the start of the intervention (PSM), we monitored participants’ complaints, symptoms, and social skills repertoire monthly, through the instruments GAD-7 (Spitzer et al., 2006), PHQ-9 (Kroenke et al., 2001), IHS-2 Del Prette (Del Prette & Del Prette, 2018), and Complaints Checklist. As of the second month of monitoring, we invited one participant per month for a more in-depth complaints assessment for case formulation purposes. We then started to apply PSM, consisting of themed sessions on 17 topics. We included a complaints and symptoms reassessment session halfway through intervention, one at the end of the intervention, and one 3 months later (follow-up). We were unable to assess participants at each session, as in Kirchner and Reis (2021), because the application of instruments on a weekly basis would demand a large portion of session time and could bias the results or impair participants’ adherence to the procedure.

Case formulation was the main tool used to customize interventions according to participants’ individual demands. The formulation models used in this study were those of Virués-Ortega and Haynes (2005) and Fonseca and Nery (2018), a more descriptive model.

Most sessions lasted between 80 and 100 min. A total of 29, 18, and 29 sessions were held with participants 1, 2, and 3, respectively, considering assessment, functional analysis, and topics. Participant 2 dropped out of the study after the intermediate evaluation. All data collection was carried out in a face-to-face format between March 2019 and March 2020.

### *Promove – Saúde da Mulher* (PSM) presentation

PSM consists of a semi-structured intervention focused especially on the development of social skills as alternative or functionally equivalent behaviors (Goldiamond, 2002; Sturmey, 1996) to those behaviors associated with the complaints of each participant, considering different contexts of interaction. This is because, when faced with interpersonal problems, socially skilled behavior tends to maximize gains and minimize harm for individuals and their community (Del Prette & Del Prette, 2017). PSM begins after screening for anxiety and depression and is composed of three major stages: the first consisting of two to four sessions for investigation and functional analysis of the complaints that prompted the participant to seek therapy; the second (and longest), consisting of 18 to 20 thematic sessions, aims to develop the socially skilled repertoire to face contingencies that lead to the disorder; the third consists of reassessing the status of complaints and reapplying standardized instruments that screened for anxiety and depression.

Themed sessions, core to the intervention, were structured as follows: investigation and discussion of spontaneous demands, discussion of homework assignments, introduction and conversation about predefined personal and interpersonal skills and their applicability in the client’s life, tips for a competent emission of themed skills, repertoire training activity, presentation of a text and a task on the discussed skills, session evaluation, and closing. After the intervention, texts were collected and are available in eBook format (Victuri & Bolsoni-Silva, 2020). This material was developed to be used as a basis for session planning, as well as to be handed out for participants to read at home.

When participants reported target behaviors for the intervention, both in spontaneous reports and in task discussions, we used contingency management (Higgins et al., 2020) and shaping (Miltenberger et al., 2020), i.e., providing more attention and/or reinforcement to the client according to how close the report was to predetermined goals. This was done gradually with increasing levels of demand according to the participant’s pace.

Discussions regarding personal and interpersonal skills and their applicability in participants’ lives started with questions such as “what is (assertiveness)?,” “what is (praising) good for?,” “how do you usually (express negative feelings)?,” “how do people react?,” and “what are the difficulties in doing that?.” From such questions, during the sessions, we carried out functional analyses (Sturmey, 1996) of target behaviors and listed alternative ways of behaving (Goldiamond, 2002; Sturmey, 1996) in more difficult situations. Suggested cues for socially competent behaviors (Del Prette & Del Prette, 2017) can be found in Victuri and Bolsoni-Silva (2020) (supplementary material to the intervention).

Repertoire building activities included selecting and role-playing (Souza et al., 2012) difficult situations associated with therapeutic goals and session topics. After role-playing, simulated situations were functionally analyzed by the participant and the therapist. According to the shaping procedure (Miltenberger et al., 2020), the therapist sought to differentially reinforce behaviors that most closely matched the therapeutic goals through positive feedback and praise. In cases of greater difficulty, the therapist also suggested adjusting behaviors presented in training, and the process was repeated until the participant achieved a performance that was more likely to be reinforced when presented in a natural environment (e.g., husband, children, work).

Handing out texts about the session skills (Victuri & Bolsoni-Silva, 2020) gave participants the opportunity to review most of the tips offered by the therapist in the session. Homework assignments, in turn, stimulated the participants to apply, observe, describe, and functionally analyze in-session behaviors in their everyday interpersonal interactions.

Topics covered in themed sessions were starting, maintaining, and ending conversations; asking and answering questions; human rights in interpersonal relationships; passive, assertive, and aggressive behavior; expressing positive feelings, praising, and thanking; giving positive feedback; giving and listening to opinions; making and responding to requests; making and responding to criticism, admitting own mistakes, and apologizing; expressing negative feelings; giving negative feedback, requesting behavior change, and negotiating; setting rules and limits; speaking in public; parenting educational practices; managing time and avoiding procrastination; and working in teams and financial behavior. Researchers followed a pre-established sequence for the three participants, organized in increasing order of difficulty according to the researchers’ experiences. Only one exception was proposed: the topic “parenting practices” was addressed earlier for participant 2 due to her frequent reports of her two children’s behavioral problems, which occurred right after the session on giving positive feedback. Each topic was approached in a way that catered for the demands of participants, according to the case formulation and the in-session reports of difficulties. For example, if a participant had more difficulty making requests during marital interactions, skill discussion and training activities were focused on the marital relationship.

Specific procedures were also used according to individual demands and clinical decision-making. For participant 1, when reports of recurring catastrophic thoughts occurred (e.g., that her child was kidnapped or had an accident), the therapist confronted her concerns with her life facts. For participant 2, when her children presented school difficulties, the therapist offered more tips on managing their behavioral problems throughout the intervention (e.g., using a token economy system and more immediate reinforcement). For participant 3, when a high rate of perfectionist behaviors occurred, the intervention included discussion and confrontation of perfectionist rules and self-rules (e.g., high housekeeping demands). For all participants, after functional analyses of anxious behaviors, the therapist discussed behavioral alternatives whenever evoking pre-aversive stimulation occurred. Recommendations for physical exercise were also offered to all participants.

### Instruments

We used the GAD-7 (Spitzer et al., 2006) and PHQ-9 (Kroenke et al., 2001) to screen for symptoms of anxiety and depression, respectively; the Del Prette IHS-2 (Del Prette & Del Prette, 2018) to assess social skills repertoires; the Complaints Checklist (developed by the authors) to monitor the status of complaints; and the Therapeutic Process Evaluation (developed by the authors) to assess participants’ satisfaction and perception of change with the intervention. Non-standardized instruments are detailed as follows. Psychometric information and detailed data for the standardized instruments can be found in Appendix 1.

The Complaints Checklist was an instrument developed by the authors based on the registration of all complaints by participants at the beginning of the research. During complaints discussion at each assessment session, the therapist uses the instrument to investigate whether there has been improvement, maintenance, or worsening in each complaint. In case there has been improvement, the participant was also asked if she would like to exclude the complaint from the intervention scope.

The Therapeutic Process Assessment was a questionnaire designed by the first author to measure participants’ satisfaction with the intervention and their perception of change throughout therapy. It consists of three open-ended questions, eight multiple-choice questions, and an open field for observations. The instrument uses self-report to assess perception of change, satisfaction with the therapy, the therapist’s handling, the degree of difficulty and suitability of the material, and participants’ degree of engagement with therapy. It was applied halfway through and at the end of intervention, as planned in its development.

### Data analysis procedures

We calculated the results of GAD-7, PHQ-9, and IHS-2 according to their normative data and statistically analyzed them using the JT method (Jacobson & Truax, 1991), on the PsicoInfo website. This method allows for statistical analysis of reliability and clinical significance of change in studies with small samples. Reliability shows if participants’ changes can be attributed to the intervention (Villa & Aguiar, 2012), whether positive or negative. In turn, clinical significance interprets whether the change represents any positive or negative impact on participants’ lives (Villa & Aguiar, 2012). Using the JT method, the results of each application here were compared with those from its previous application carried out with the same participant.

The JT method uses data from larger samples as bases for analysis, which can be obtained from instrument validation studies. Reference samples were found in Johnson et al. (2019) for GAD-7, Ferreira et al. (2017) for PHQ-9, and Del Prette and Del Prette (2018) for IHS-2. Some data released in the analysis of the IHS-2 Del Prette that were not included in its manual were shared by Dr. Zilda Aparecida Pereira Del Prette.

The Complaints Checklist analyzed each complaint quantitatively, and a score was assigned for each status: zero points for withdrawn, one for improvement, two for stability, and three for worsening. In the Therapeutic Process Evaluation, we quantitatively analyzed multiple-choice questions and qualitatively analyzed open-ended questions through the analysis of responses in mutually exclusive categories.

## Results

PSM brought positive results for all participants, with emphasis on anxiety and depression levels. Some improvements in social skills were evidenced by the instrument and others by participants’ reports. Most complaints were resolved and participants felt satisfied with the intervention.

As participant 2 dropped out of the study after the intermediate assessment phase, we were not able to assess the stability of positive changes. Despite her interest, her elevated absence rate from therapy sessions kept her from further participating. In addition to working, doing household chores, and looking after her children, she was studying for an undergraduate degree. When the internship period began, she stated she would no longer be able to attend sessions. We offered online sessions, but she also did not attend. As her initial complaints lowered by the intermediate assessment—including symptoms of anxiety and depression—we believe her motivation to continue was compromised.

### Anxiety and depression

**Fig. 1:**
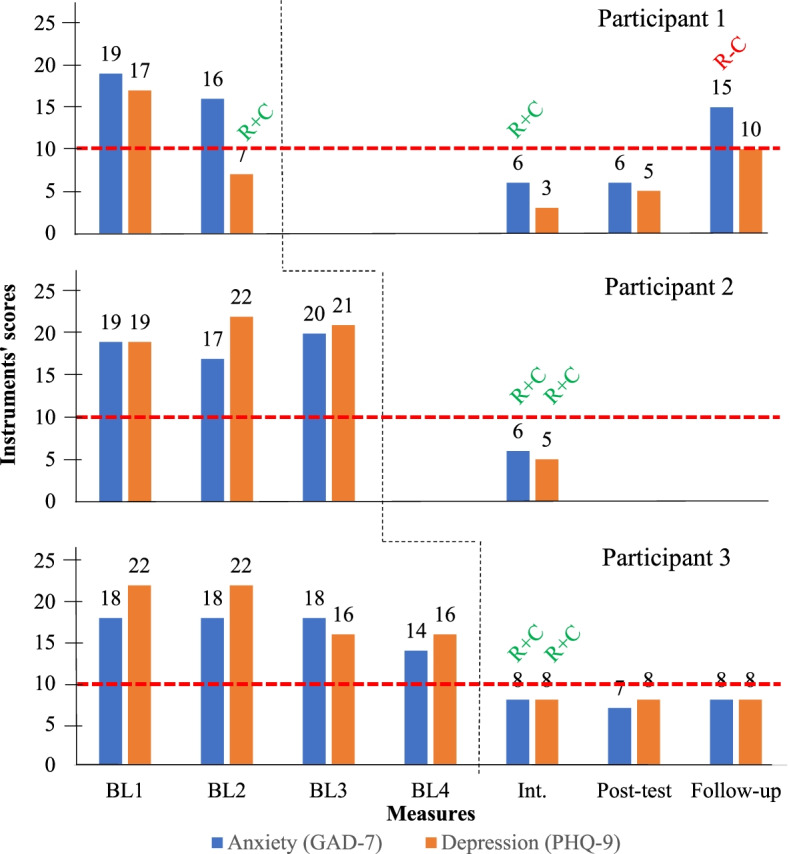
Anxiety (GAD-7) and depression (PHQ-9) results. *Note*: BL = baseline; R+C = reliable positive change; R-C = reliable negative change; red dotted line = instruments cut score; black dotted line = beginning of the intervention for each participant

Figure 1 shows the results for GAD-7 and PHQ-9 throughout the study.

The first half of the intervention sufficed to turn the participants’ scores to non-clinical level at the intermediate measure, what was also a reliable change according to JT method, despite participant 1’s level of depression, which was no longer clinical at baseline 2. Therefore, this improvement cannot be attributed to the intervention and constitutes a methodological limitation of this study.

Participant 1 remained outside the clinical level for anxiety and depression until posttest. However, she showed a reliable relapse in anxiety levels at follow-up. Regarding cutoff scores, she returned to clinical levels for anxiety and depression 3 months after concluding the intervention, accounted for by an extraneous factor. Her husband struggled to deal with her increased levels of assertiveness, which furthered the frequency and intensity of his control over the home environment and caused the participant to show increased anxious and depressed behaviors. As the issue was more focused on her husband, we initially referred him to individual therapy, psychiatric care, and, later, couples therapy.

Participant 3 kept non-clinical scores for both disorders with no new reliable changes until the last assessment performed 3 months after the end of the intervention. This consisted of the most successful case of collected evidence for the present study as it showed the greatest stability at baseline and stability of gains after the end of therapy.

Due to their heterogeneity, data from instruments’ reference samples made it impossible for the JT method to identify clinically significant changes. However, the cutoff scores allowed us to analyze the relevance of such changes.

### Social skills

**Fig. 2:**
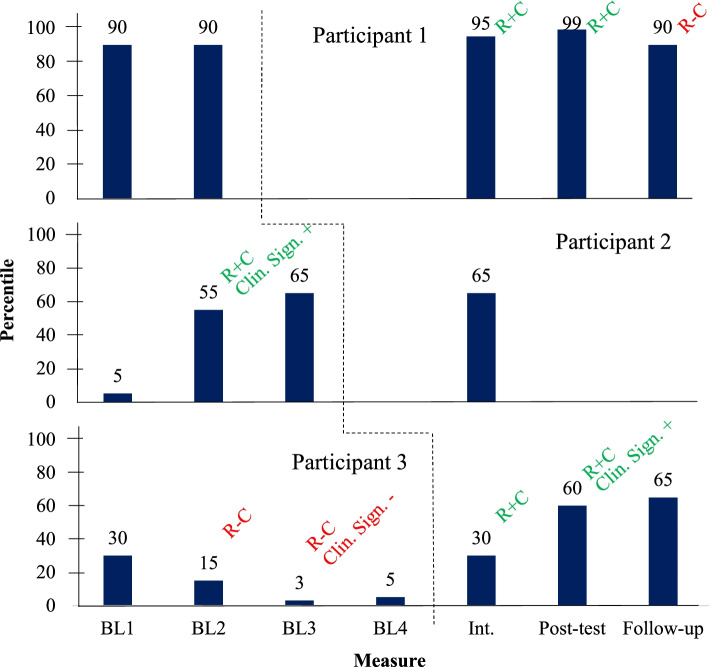
Social skills total scores percentiles (IHS-2 Del Prette). *Note*: BL = Baseline; R+C = reliable positive change; R-C = reliable negative change; Clin. Sign. + = clinically significant positive change; Clin. Sign. - = clinically significant negative change; black dotted line = beginning of the intervention for each participant

Figure 2 describes participants’ total scores in terms of their percentiles according to the reference population used to validate the instrument. The JT method was able to identify reliable and clinically significant changes.

Participant 1 showed high levels of social skills throughout the entire study. Even so, we were able to observe reliable positive changes at intermediate and posttest assessments. At follow-up, however, there was a reliable worsening with return to baseline levels. We believe this is due to her perceived ineffectiveness to deal with marital conflicts presented at that time. Nevertheless, she maintained very elaborate repertoires according to the instrument’s classification.

We hypothesize that this may be due to a limitation of the instrument in measuring the repertoire targeted by the program and/or may have occurred as an effect of the punishment applied by the participant’s husband on her self-report, such that she rated her repertoire more negatively as a consequence of her husband punishing her behavioral changes.

Participant 2 showed a reliable and clinically significant positive change in her social skills repertoire between the first and second baseline assessments. We hypothesize that this major change during a period without intervention is probably due to her lack of understanding of the instrument at first. After the second application, she showed no more reliable or clinically significant changes, which points to the stability of her repertoire at a level slightly above average.

Participant 3 showed reliable repertoire worsening during baseline, one of which with clinical significance. Successive applications may have increased this participant’s perception of her behavioral deficits. After starting the intervention, she showed two reliable positive repertoire changes, one of which was clinically significant. At follow-up, an improvement was observed in relation to posttest, but without statistical relevance. This participant also ended the study with a level slightly above average of social skills according to the instrument.

### Complaints

**Fig. 3:**
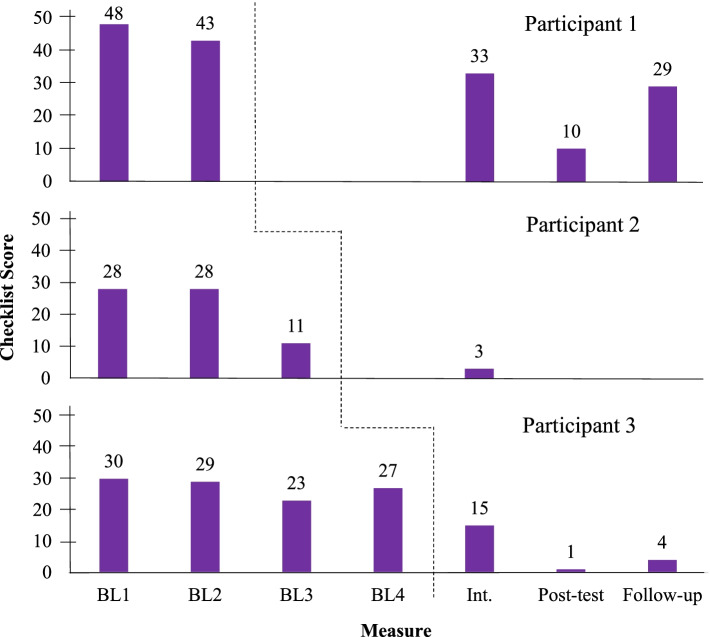
Complaints Checklist Scores. *Note*: BL = baseline; black dotted line = start of intervention for each participant

The Complaints Checklist scores obtained by participants in each assessment session are shown in Fig. 3.

Regarding baseline, participants 1 and 3 kept most of their complaints throughout assessment sessions prior to intervention, most of them related to interactions with their children and husband. Participant 2, in turn, did not report many of her complaints in baseline assessment 3, especially those related to work. In turn, participants 1 and 3 presented one complaint at most about work throughout the entire study. Therefore, interpersonal relationships in this context were not the focus of any interventions.

At intermediate assessment, participant 1 showed a slightly decreased score at the Checklist, while participants 2 and 3 showed a greater number of improvements. In the posttest assessment, there was an improvement in many of participant 1’s complaints and withdrawal of almost all participant 3’s complaints. The only one remaining was a complaint of insecurity, but with improvement. At follow-up, participant 1 recovered most of her husband-related complaints, and participant 3 recovered one previously withdrawn complaint.

### Client’s satisfaction

In multiple-choice questions from the Therapeutic Process Assessment, participants regarded the therapist as very skilled to help them, the therapist’s attitude as adequate, the tasks and texts as consistent with their goals, and their own engagement in therapy and tasks as high. In questions assessing the difficulty of tasks, participants 1 and 3 reported difficulties. However, both were able to perform and deliver all required tasks. This can be because necessary repertoire changes to solve long-term problems require confronting highly aversive contingencies, such as asking the children for help with the housework or defending one’s rights in front of their husband and confronting important rules, such as not valuing someone’s performance when they do nothing beyond their duty. In the question regarding text difficulty, only participant 2 ranked it as average, but without significant difficulty in understanding. The others ranked texts as easy.

The answers to open-ended questions of the questionnaire revealed three categories of gains from the procedure perceived by participants, the first two being the most frequent: solution of problems/difficulties, self-awareness, and increased quality of life. The solution of problems and difficulties (“I can manage to act more assertively while interacting with people and while dealing with my feelings”; participant 3) focused on interpersonal relationships, while self-knowledge (“I learn to know myself and resolve internal conflicts I have”; participant 1) and increased quality of life (“it improved my thoughts and routine”; participant 2) were more noted in the personal sphere.

## Discussion

Gender inequality, reported by Correia and Borloti (2011) as a risk factor for anxiety and depression, appeared in all three cases, especially regarding childcare and household chores. The responsibility for these tasks was mostly assigned to the participants with little to no participation of their husbands.

Inequality in task distribution overloaded participants’ routines and consumed time that could be invested in rest and leisure activities, in addition to generating pending issues that made them anxious. Throughout the intervention, they benefited from us helping them negotiate and value the participation of their children and husbands in these tasks.

The improvement in anxiety and depression observed here is comparable to those from other interventions with similar aims (Kladnitski et al., 2018; Mahan et al., 2018; Păsărelu et al., 2016). We were able to adapt PSM to meet the functional particularities of difficulties faced by each participant. This process of adaptating and setting priorities was greatly facilitated by case formulation, as defended by Sturmey (2014). Schwartz et al. (2019) also used a relatively structured behavioral treatment and observed reduced anxiety and depression in participants.

Also, regarding anxiety and depression outcomes, we noticed that the instruments showed no more clinical levels in intermediate assessments for all participants. The first half of the intervention sufficed to achieve this change, despite participant 1’s level of depression, which was no longer clinical at baseline. This data suggests that an abridged version of PSM can achieve the same results in a shorter time, as suggested by the American Psychological Association (2006).

Understanding the risk factors for the development of disorders made the authors turn their attention to important elements present in participants’ lives—task overload due to gender inequalities (Correia & Borloti, 2011) and interpersonal difficulties (Starr et al., 2014) being the main ones. Most of the topics on PSM are related to interpersonal relationships that overloaded participants’ lives. We believed that the first step toward lifting this burden was to understand how these relations occurred. After that, they had to present behaviors compatible with a long-term solution of problems in order to establish more balanced relationships.

Participant 1’s case faced an important limitation: her difficulty in directly addressing her husband’s psychological problems. Also, we were not able to predict her husband’s retaliation through the case formulation, which can be considered as a methodological limitation of the present study and may be considered in future studies. Due to a relapse identified in the follow-up session, the participant required continued individual therapy for approximately 1 year (with gradual withdrawal until discharge), her husband was referred to psychiatric care, and both received couples therapy for about 6 months. We believe that individual therapy for participants 2 and 3’s husbands could also have been beneficial, but they showed no opening for such. Future studies may include interventions for the husbands, in case there are similar demands.

Participant 1’s improvement can be explained mainly by the development of an alternative repertoire to deal with pre-aversive stimuli (Dymond & Roche, 2009; Skinner, 1953; Zamignani & Banaco, 2005) and by the development of a more adaptive repertoire to obtain reinforcers (Abreu & Abreu, 2017), whether positive—such as affection from her children and husband—or negative—such as problem-solving. Participant 2’s improvement is attributed to her leaving a job in which there was an excess of signaled (Coêlho & Tourinho, 2008) and uncontrollable (Dougher & Hackbert, 1994) punishment and the development of a more adaptive repertoire to obtain important reinforcers (Abreu & Abreu, 2017) in the relationship with her children and husband. For participant 3, we believe there was a development of an alternative repertoire to deal with pre-aversive stimuli (Dymond & Roche, 2009; Skinner, 1953; Zamignani & Banaco, 2005) associated with task overload and development of alternative repertoires to achieve affection and help in household chores—both strong reinforcers for her (Abreu & Abreu, 2017; Goldiamond, 2002; Sturmey, 1996), which were scarce before therapy and produced uncontrollable aversive accumulation (Dougher & Hackbert, 1994) of tasks.

The social skills instrument does not assess the outcomes obtained by repertoire gains and, also, is not able to specify in which contexts (e.g., marital, parental, work) the social skills are presented. However, participants’ reports throughout the intervention point to positive consequences of these changes, indicating an increase in social competence (Del Prette & Del Prette, 2017).

We believe that by expanding socially skilled repertoires, positively reinforcing contingencies took place in participants’ interpersonal relationships. Such contingencies replaced those that maintained behavioral deficits and excesses, usually characterized as anxiety and depression disorders. This hypothesis is corroborated by fewer reports of difficulties in interpersonal interactions (e.g., task overload, aggressive arguments with children and husband, conflicts at work), coupled with an increase in reports of conflict resolution (e.g., redistribution of household chores) and positively reinforcing interactions (e.g., exchanges of affection and leisure activities with children and husband, proximity to clients or co-workers). Thus, some repertoire improvements, more clearly observed in participant 3, indicate that PSM influenced social skills repertoires. Future studies may include specific and contextual measures for social skills repertoire, as to allow the researchers to better assess the participants’ deficits since the first sessions and improve the adaptation of the intervention.

Results from the Complaints Checklist pointed to the reduction of interpersonal problems for the three participants, especially in marital and parental contexts. There was also a reduction in physical symptoms such as increased heartbeat and sweating, which are compatible with anxiety and depression disorders according to the DSM-V (American Psychiatric Association, 2013) and were reported as complaints. The intervention was applied among women with problems across multiple contexts of their lives, which consist of more complex cases with a worse prognosis (Cyranowski et al., 2012; Fernandes et al., 2018), but we believe that it can also be adapted and geared to women with problems in specific contexts.

Seeking to analyze how well the PSM program meets the efficacy criteria, critical to evidence-based practice (American Psychological Association, 2006), we analyzed which of the 17 criteria for evaluating the efficacy of psychological interventions, as pointed out by Durgante and Dell’Aglio (2018), were met. We consider that 11 of them were met, listed in Appendix 2. Regarding the efficacy statement (Durgante & Dell’Aglio, 2018), considering this study’s results and limitations, PSM appears to have been efficacious in lowering depression and anxiety during its application, improving social skills and/or social competence, and reducing complaints about women with anxiety and depression symptoms, as well as interpersonal problems in different areas of their lives.

The other criteria include a broad analysis of relevant empirical literature (due to the lack of studies in TAC for the public), systematic clinical observation and consensus among recognized experts, sampling techniques and use of a control group, need for other studies with the same intervention program, and detailed description to enable replications. These criteria were not met by the present study, however, we believe they can be contemplated in further studies with PSM.

Satisfaction outcomes address an evidence gap in TAC-based treatments (Moraes & Silveira, 2019). Such data are comparable to those of Kladnitski et al. (2018), who used cognitive behavior therapy and mindfulness combined. This demonstrates TAC’s potential to address the third element of evidence-based practice.

From positive outcomes, we highlight improvements in anxiety and depression by the first half of the intervention, lowered reported complaints, and clients’ satisfaction with therapy and the therapist’s conduct. From negative outcomes, we highlight participant 2’s dropout after the intermediate assessment and participant 1’s relapse at follow-up.

## Conclusions

Our study sought to describe the effects of PSM on the participants’ indicators of anxiety, depression, behavior, complaints, and satisfaction, thus adding preliminary evidence to a TAC intervention. In this sense, we believe to have achieved such goals. We showed promising results that justify further research using PSM.

Because this is the first undertaking for the development of an intervention manual, we have been unable to meet all efficacy criteria. Therefore, the present study can be considered a feasibility or pilot study for future research with PSM.

Important limitations were the improvement in participant 1’s depression score before the intervention, the dropout of one participant halfway through the intervention, low baseline stability for social skills, and difficulty to meet the suggested criteria for multiple baseline designs, especially the number of instrument applications. Given these limitations in an initial study, we suggest aiming to overcome them in future studies.

Future studies should include instruments that assess specific social competence for parenting and conjugality. We also suggest replications with more participants, in different locations, with experimental designs and more applications of anxiety and depression instruments throughout the intervention. Including a survey that looks at the effects of an abridged version of the program would also be useful to lower the number of sessions per client.

## Data Availability

The datasets used and analysed during the current study are available from the corresponding author on reasonable request.

## Appendix 1

### Psychometric information for the standardized instruments used

Table 1

**Table 1:** Instruments’ information for GAD-7, PHQ-9, and IHS-2 Del Prette

Instrument	Construct	Psychometric properties
GAD-7 (Spitzer et al., 2006)	Anxiety	*α* = 0.916; *ρ* = 0.909 (Moreno et al., 2016)
PHQ-9 (Kroenke et al., 2001)	Depression	Sensitivity = 1.00 Specificity = 0.98 Positive predictive value = 0.97 Negative predictive value = 1.00 (Santos et al., 2013)
IHS-2 Del Prette (Del Prette & Del Prette, 2018)	Social skills repertoire	*α* = > 0.770 (Del Prette et al., 2021)

## Appendix 2

### Evidence-based practice criteria met

Table 2

**Table 2:** Efficacy criteria (Durgante & Dell’Aglio, 2018) met by the present study

Criteria	Parts of the text in which the criteria were met
3. Guidelines should specify what outcomes the intervention aims to produce and the required evidence for each outcome.	PSM description Results
4. It is important to consider the method and rationale for selecting participants as well as how the sample represents the population (group equivalence) and phenomena of interest.	Criteria for selection of participants
6. Measures used to evaluate the results must have psychometric validity.	Appendix 1
8. An efficacy statement should be formulated that “program or policy X is efficacious in producing outcomes Y for population Z.”	Discussion
9. Individual pre-test differences should be evaluated.	Brief case formulations
10. For long-term results, there should be at least one follow-up.	Method Results
11. All results should be displayed (positive, non-significant, or negative).	Results
12. Statistics should be used to ensure unbiased estimates in the results.	Data analysis procedures for standardized instruments
13. Results should consider the clinical significance of the intervention.	Data analysis procedures Results for standardized instruments
15. Results should contain the main components of the intervention, time to observe effects, and mediating or moderating variables	Results
17. The study should assess and report rates of losses and dropouts, which may compromise the generalizability of the results to other samples/populations.	Results Discussion

Criteria translated from Durgante and Dell’Aglio (2018)

## Abbreviations

PSM: *Promove – Saúde da Mulher*
TAC: Terapia Analítico-Comportamental (clinical behavior analysis)

## Footnotes

1 For the present study, the term TAC will be used as a synonym of behavior therapy (Goldiamond, 2002; Skinner, 1953; Sturmey et al., 2020), and clinical behavior analysis (Moraes & Silveira, 2019; Zettle, 2020).
